# Polycystic ovarian syndrome is accompanied by repression of gene signatures associated with biosynthesis and metabolism of steroids, cholesterol and lipids

**DOI:** 10.1186/s13048-015-0151-5

**Published:** 2015-04-13

**Authors:** Dessie Salilew-Wondim, Qi Wang, Dawit Tesfaye, Karl Schellander, Michael Hoelker, Md Munir Hossain, Benjamin K Tsang

**Affiliations:** Institute of Animal Science, Animal Breeding and Husbandry Group, University of Bonn, Endenicher Allee 15, Bonn, 53115 Germany; Reproductive Biology Unit and Division of Reproductive Medicine, Department of Obstetrics & Gynecology and Cellular & Molecular Medicine, Interdisciplinary School of Health Sciences, University of Ottawa, Ottawa, K1H 8L6 ON Canada; Chronic Disease Program, Ottawa Hospital Research Institute, The Ottawa Hospital (General Campus), Critical Care Wing, 3rd Floor, Room W3107, 501 Smyth Road, Ottawa, K1H 8L6 ON Canada; Department of Animal Breeding and Genetics, Bangladesh Agricultural University, Mymensingh, 2202 Bangladesh; Department of Agricultural Biotechnology, World Class University Major in Biomodulation, College of Agriculture and Life Sciences, Seoul National University, Seoul, 151-921 South Korea

**Keywords:** Polycystic ovarian syndrome, Anovulation, Gene expression, Ovary

## Abstract

**Background:**

Polycystic ovarian syndrome (PCOS) is a spectrum of heterogeneous disorders of reproduction and metabolism in women with potential systemic sequel such as diabetes and obesity. Although, PCOS is believed to be caused by genetic abnormalities, the genetic background that can be associated with PCOS phenotypes remains unclear due to the complexity of the trait. In this study, we used a rat model which exhibits reproductive and metabolic abnormalities similar to the human PCOS to unravel the molecular mechanisms underlining this complex syndrome.

**Methods:**

Female Sprague–Dawley rats were randomly assigned to DHT and control (CTL) groups. Rats in the DHT group were implanted with a silicone capsule continuous-releasing 83 μg 5α-dihydrotestosterone (DHT) per day for 12 weeks to mimic the hyperandrogenic state in women with PCOS. The animals were euthanized at 15 weeks of age and the pairs of ovaries were excised and the ovarian cortex tissues were used for gene expression analysis. Total RNA was from the ovarian cortex was amplified, labeled and hybridized to the Affymetrix GeneChip® Rat Genome 230 2.0 Array. A linear model system for microarray data analysis was used to identify genes affected in DHT treated rat ovaries and the molecular pathway of those genes were analyzed using the Database for Annotation, Visualization and Integrated Discovery (DAVID) analysis tool.

**Results:**

A total of 573 gene transcripts, including *CPA1, CDH1, INSL3, AMH, ALDH1B1, INHBA, CYP17A1, RBP4, GAS6, GAS7* and *GATA4,* were activated while 430 others including *HSD17B7, HSD3B6, STAR, HMGCS1, HMGCR, CYP51, CYP11A1 and CYP19A1* were repressed in DHT-treated ovaries. Functional annotation of the dysregulated genes revealed that biosynthesis and metabolism of steroids, cholesterol and lipids to be the most top functions enriched by the repressed genes. However, cell differentiation/proliferation, transcriptional regulation, neurogenesis, cell adhesion and blood vessel development processes were enriched by activated genes.

**Conclusion:**

The dysregulation of genes associated with biosynthesis and metabolism of steroids, cholesterol and lipids, cell differentiation/proliferation in DHT- treated ovaries could be a molecular clue for abnormal steroidogenesis, estrous cycle irregularity, abnormal folliculogenesis, anovulation and lipid metabolism in PCOS patients.

**Electronic supplementary material:**

The online version of this article (doi:10.1186/s13048-015-0151-5) contains supplementary material, which is available to authorized users.

## Introduction

The ovary is a key organ in the female reproductive system and its malfunction due to endocrine abnormalities could result in female infertility. Polycystic ovarian syndrome (PCOS) is one of the common hormonal and metabolic disorders in women of reproductive age [1]. However, due to its heterogeneity and complexity, universally accepted clinical definition of PCOS remains ambiguous [2,3]. Indeed, the presence of polycystic ovarian morphology is one of the common phenomena that can occur in the majority of PCOS patients. About 95% of women with PCOS at their early follicular phase could have polycystic ovaries and reduced level of follicle stimulating hormone [4] which may lead to antral follicle growth arrest and increased luteinizing hormone level [5]. In addition, PCOS is also associated with hyperandrogenism, menstrual dysfunction, oligo-ovulation and insulin resistance [6]. In this context, PCOS is considered as a complex androgen excess accompanied by different degrees of gonadotropic and metabolic dysregulation controlled by multiple gene interaction and environmental factors [7]. However, to what extent this trait is transmitted to the next generation and the intrinsic molecular factors underlining the occurrence of PCOS is unclear.

Although, the genetic basis of abnormal follicular development, anovulation, metabolic disorder and other heterogenous clinical abnormalities of PCOS patients seems to require detailed investigation, it is suggested that daughters from women exhibiting a characteristics of PCOS could have a higher chance of acquiring hyperandrogenism and other PCOS phenotypes [8]. Moreover, single nucleotide polymorphism in thyroid adenoma associated (*THADA),* DENN/MADD domain containing 1A (*DENND1A)*, interleukin 6 (*IL6)* and adiponectin genes has been suggested to be the genetic causes of PCOS [9-11]. In addition, in vitro studies also showed altered expression of *CYP11A* and *CYP17* genes in theca cell derived from PCOS woman [12]. Furthermore, changes in the granulosa and theca cell gene expression have been reported in women with PCOS [13-15]. Although these association studies were performed using the samples of PCOS patients, the majority of gene expression studies were based on the cell culture models which may not necessarily represent and describe the biological and molecular networks governing its complex phenotype. Indeed, this can in part be due to the availability and accessibility of the human sample or small sample size of the study populations [7] and the complexity of the trait between individuals. However, to addresse the clinical heterogeneity of PCOS, animal models have been described to be the best option to investigate the pathophysiologic mechanisms associated with the etiology of PCOS [16-21]. Therefore, to uncover the broad basis of molecular mechanisms associated with physiological and anatomical changes induced by PCOS, we generated a rat PCOS model that exhibit both polycystic ovaries (PCO) and metabolic abnormalities by implanting silastic capsules containing 5α-dihydrotestosterone (DHT) into their ovary in similar way as previously described by others [22]. Using this rat PCOS model that exhibits both polycystic ovaries (PCO) and metabolic abnormalities, we have previously demonstrated altered expression of 89 miRNAs following chronic androgen treatment [23]. However, the genes that are activated or repressed as well as their molecular functions, gene networks and molecular pathways associated with PCOS phenotypes, remained unclear. Therefore, this study was conducted to gain insight into the genes that are associated with follicular arrest, abnormal steroid and metabolite biosynthesis and metabolism, insulin resistance and ovarian dysfunction.

## Materials and methods

Details of the materials and methods used in the present study have been described in our previous publication [23]. Briefly, female Sprague–Dawley rats were randomly assigned to DHT and control (CTL) groups. Rats in the DHT group were implanted with a silicone capsule continuous-releasing 83 μg 5α-dihydrotestosterone (DHT) per day for 12 weeks to mimic the hyperandrogenic state in women with PCOS, whose plasma DHT levels are approximately 1.7-fold higher than those of healthy control and those in CTL group received empty capsule [22]. The animals were euthanized at 15 weeks of age, ovaries were excised and extraneous tissues carefully removed. Corpus luteum (CL) was present in most of control rat ovaries while none or very few CL were observed in DHT-treated rat ovaries. Ovarian cortex tissues were snap-frozen in liquid nitrogen and stored at −80°C for further analysis. The PCOS phenotypic characteristics of DHT-treated rats have been described [23].

### Gene expression analysis using GeneChip@rat genome array

Total RNAs were isolated from 3 independent DHT and CTL rat ovaries using miRNeasy mini kit (Qiagen, Hilden, Germany). Genomic DNA contamination was removed from the RNA samples using TURBO DNA-free™ kit (Ambion, Foster City, CA). The concentration of the RNA was analyzed using the Nanodrop 8000 Spectrophotometer (Thermo Fisher Scientific Inc, DE, USA). The RNA integrity and quality was evaluated using Agilent 2100 Bioanalyzer with RNA 6000 Nano LabChip® Kit (Agilent Technologies Inc, CA, USA).

### RNA amplification

250 ng of total RNAs from DHT-treated or CTL rat groups in four replicates was amplified and labeled as per the GeneChip®3’ IVT Express Kit (Affymetrix, CA, USA). Eukaryotic poly-A RNA control kit (Affymetrix, CA, USA) was used as a SPIKE-IN control to monitor the entire target labeling process. Following amplification, the biotin labeled amplified RNA (aRNA) was purified and fragmented. The distribution of aRNA fragments were evaluated using Agilent 2100 bioanalyzer with RNA 6000 Nano LabChip® Kit (Agilent Technologies Inc, CA, USA).

### Sample hybridization, array washing, staining and scanning

Prior to hybridization, the fragmented and biotin labelled cRNA from each rat ovary was mixed with control oligonucleotide B2 (3 nM), 20× eukaryotic hybridization controls (bioB, bioC, bioD, cre) (Affymetrix, CA, USA), 2× hybridization mix and DMSO. The hybridization cocktail were then incubated at 99°C (5 min) and subsequently at 45°C (5 min). Each sample was then transferred to independent GeneChip® Rat Genome 230 2.0 Array chip. Three biological replicates and one technical replicate (pool of three biological replicates) were hybridized for each rat group for 16 h. The array slides were washed and stained using the Fluidics Station 450/250 (Affymetrix, CA, USA), according to the GeneChip® expression user manual (P/N 702232 Rev. 3). Arrays were scanned with the GeneChip™ 3000 laser confocal slide scanner (Affymetrix, CA, USA) integrated with GeneChip® Operating System (GCOS).

### Array data analysis and visualization

The array data was normalized by integrating the bioconductor packages (http://bioconductor.org) in R environment (www.r-project.org), using GC robust multi-array average analysis [24]. Briefly, the cell intensity **(**CELL**)** files were imported into R software after loading bioconductor packages (http://bioconductor.org) that suit to the Rat GeneChip affymetrix array. The normalized data and the CELL files are stored in the Gene Expression Omnibus (GEO; http://www.ncbi.nlm.nih.gov/geo/, series entry number GSM1437398). The linear models for microarray data analysis system (LIMMA) [25] and the Benjamini–Hochberg procedure of false discovery rate adjustment [26] were employed to discriminate the gene expression profile between the samples. The differentially expressed genes were tested for their gene ontology (GO) terms for over- or under-representation, using a classical hypergeometric test [27]. The molecular pathway enriched by differentially expressed genes were obtained from the Kyoto Encyclopedia of Genes and Genomes (KEGG) and Panther pathway data bases, using The Database for Annotation, Visualization and Integrated Discovery (DAVID) analysis tool [28]. The heatmaps and clustering of differentially expressed genes were constructed using Bioconductor (http://www.bioconductor.org) in R software environment http://www.r-project.org/ and/or PermutMatrix [29].

### Validation of differentially expressed genes

Some of the differentially expressed genes were randomly selected for validation, using SYBR Green based quantitative real time polymerase chain reaction (qPCR) and with sequence specific primers designed online (http://frodo.wi.mit.edu/primer3/; Table 1). The specificity and identity of each primer pair was confirmed by sequencing with the GenomeLab™ GeXP Genetic Analysis System (Beckman Coulter). The mRNA level was subsequently quantified using the cDNAs obtained from reverse transcription of total RNA samples used for microarray study. The qPCR was then performed in 20 μl reaction volume containing iTaq SYBR Green Supermix with ROX (Bio-Rad laboratories, Munich, Germany), the cDNA samples of DHT or CTL samples and the specific forward and reverse primer in the StepOnePlus™ Real-Time PCR Systems (Applied Biosystems, Foster City, CA). The presence of specific amplification was monitored by evaluating the dissociation curve. The abundance of each transcript was determined using the comparative threshold cycle (ΔCT) method. Data were analyzed after normalizing the Ct value of the target genes against the housekeeping gene β actin (β-actin). The Student’s *t*-test or least significant difference test procedures was employed to detect differences in mRNA levels between samples. The level of activation or repression of a gene in DHT relative to CTL was determined using the formula 2^-**ΔΔC**T^. Differences with p < 0.05 were considered significant.

**Table 1:** **Gene specific primers used for validation of differentially expressed genes**

**Gene bank acc. No**	**Gene symbol**	**Primer 5’ to 3’**	**bp**
**NM_012536**	**CTRB1**	**F: CTGAAGATCGCACAGGTCTTT**	**185**
		**R: TCTTGAGGGCATTGTATTTGG**	
**NM_017239**	**MYH6**	**F: AAGCTGCAGTTGAAGGTGAAG**	**214**
		**R: TGGACAGGTTATTCCTCATCG**	
**NM_013085**	**PLAU**	**F: GAGGGTGCTTGTCCAATATGA**	**189**
		**R: CAGGAATACACCAGCTTTGCT**	
**NM_207602**	**ST3GAL6**	**F: TGCGTATCACAATCTGACTGC**	**200**
		**R: AATCACCAGGCAGCAACAG**	
**NM_017128**	**INHBA**	**F: TAGGCAGTCTGAAGACCATCC**	**199**
		**R: TGAGTGGAAGGAGAGTGAGGA**	
**NM_031558**	**STAR**	**F: CTCACGTGGCTGCTCAGTAT**	**221**
		**R: CTTGGCTGAAGGTGAACAGA**	
**NM_017235**	**HSD17B7**	**F: CTTTTAGTCCCAGCGAGGAG**	**188**
		**R: TGGCCCAAACACAAACATAC**	
**NM_138504**	**OSGIN1**	**F: CAATCCCTGAGGAGGAAGAG**	**217**
		**R: CCCCTCTGGTCTATGGCTAC**	
**NM_013413**	**RLN1**	**F: CGTTCCCAGAGCTACAACAAC**	**249**
		**R:CCATTAGCTCCGTATCAGCAG**	
**NM_031144**	**ACTB**	**F: ACTGGGACGATATGGAGAAGA**	**202**
		**R:AGAGGCATACAGGGACAACAC**	

(bp = number of base pairs).

## Results

### Ovarian polycystic syndrome is associated with dysregulation of gene expression

**Figure 1:**
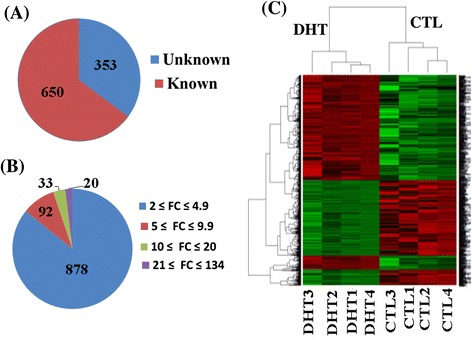
Ovarian transcriptome profile differences between DHT-treated and CTL rat groups. **(A)** The number of annotated (with known gene symbol) and non-annotated (unknown gene symbol) dysregulated genes in DHT rat groups. **(B)** The fold change (FC) distribution of dysregulated genes. **(C)** The heatmap showing the expression pattern of differentially expressed genes within and between biological replicates. DHT represents biological replicates in DHT-treated rat groups while CTL represents biological replicates in rats received an empty silastic capsule. Heatmap was generated using normalized log_2_ transformed values and the normalized log_2_ transformed expression values are described by pseudo color scale with red indicating the activated transcript level while the green color shows repressed expression pattern of a specific gene.

This study was a part of our previously published results and thus a detailed description of the PCOS phenotypic data has been provided [23]. The DHT- treated rats had higher body weight, oestrous cycle irregularity and reduced insulin sensitivity. Ovaries from these rats had lower weight and exhibited absence of corpus luteum, higher percentage of follicle cysts, relatively thin membrane granulosa and theca hyperplasia. Thus, to unravel the genes that are abnormally activated or repressed due to PCOS, the ovarian transcriptome profile of DHT-treated and CTL rats were compared using the GeneChip @rat Genome array. The genes of which the pattern of expression was different between the DHT and CTL groups were identified by a linear model described in the LIMMA pipeline and bioconductor packages. 1774 of the 31046 gene transcripts available in GeneChip® Rat Genome 230 2.0 A array showed ≥ 2 fold change dysregulation in DHT-treated rat ovaries compared to CTL group (data not shown). However, using a stricter selection criteria (fold change ≥ 2, p ≤ 0.01 and false discovery rate (FDR) ≤ 10%), a total of 1003 transcripts were dysregulated in DHT-treated compared to CTL group (Figure 1A and B). Hierarchical clustering of the dysregulated genes exhibited a similar expression pattern within biological replicates and distinct differences between the groups (Figure 1C).

### Gene transcripts repressed in DHT-treated rats

**Figure 2:**
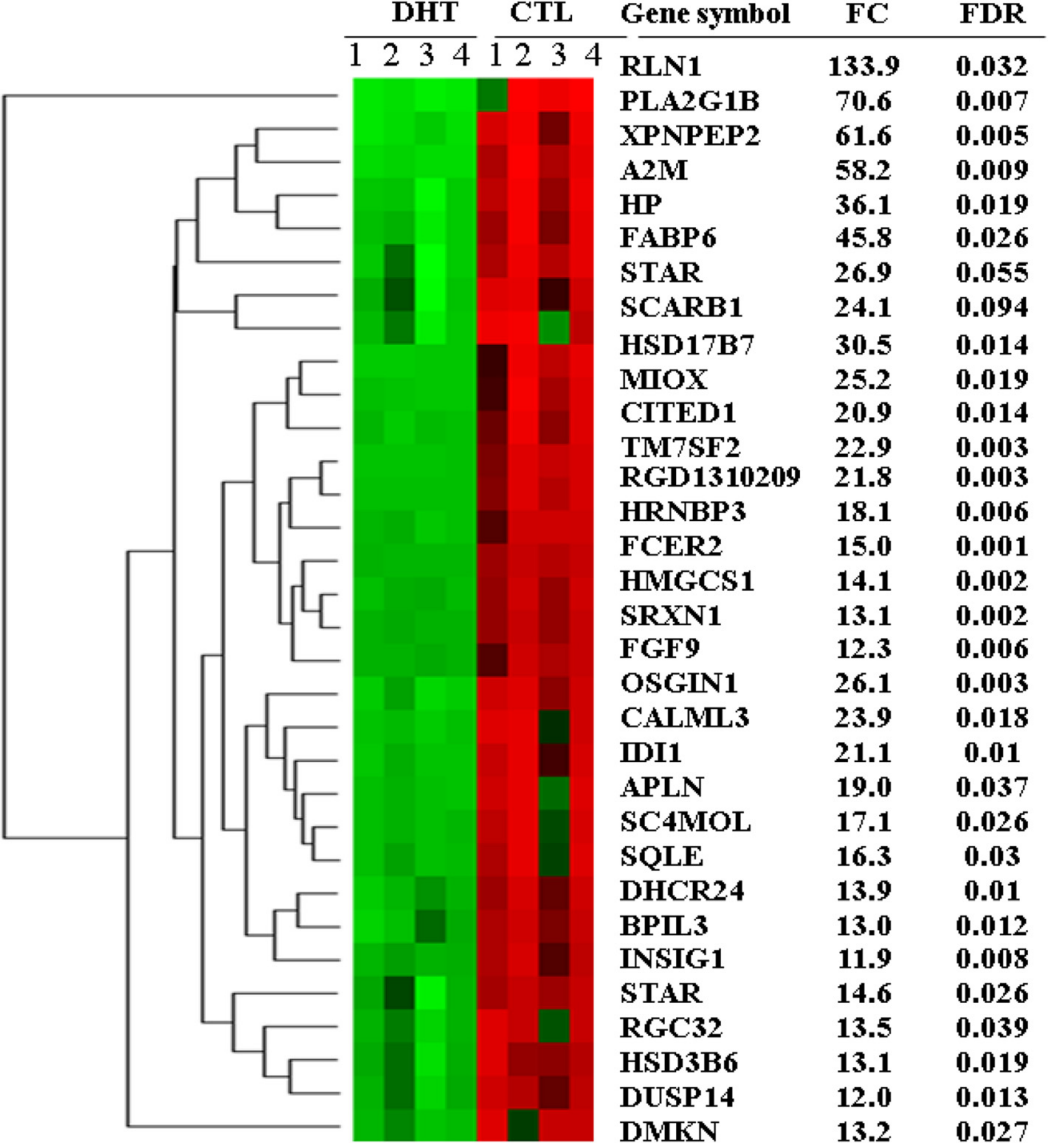
Significantly repressed genes by ≥ 11.9 folds in DHT-treated rat ovaries. The hierarchical clustering, the expression pattern, the average fold change (FC) and the false discovery rate of significantly highly repressed genes in DHT treated rat ovaries. DHT represents biological replicates in DHT-treated rat groups while CTL represents biological replicates in rats received an empty silastic capsule. The red color indicates the activated transcript level in CTL groups while the green color shows repressed expression pattern of a specific gene in DHT treated rat ovaries.

Following identification of differentially expressed genes between DHT-treated and CTL groups, the genes of which their transcript level was significant reduced by ≥ 2 folds changes in DHT compared to CTL were considered as repressed genes. However, those genes which showed by ≥ 2 folds reduction but displayed a false discovery rate higher that 0.1 were excluded in the analysis. Based on these stringent criteria, including 123 express sequence tags (ESTs), a total of 430 gene transcripts were found to be repressed in the DHT-treated ovaries. Of all the repressed genes, 320 transcripts displayed fold changes between 2 and 5 whereas the expression level of 110 genes was dysregulated between 5 and 134 folds in the DHT group. The list and expression pattern of genes repressed by 11.9 or more folds are described in Figure 2. Among the top repressed genes, the expression pattern of relaxin 1 (RLN1), Phospholipase A2 (*PLA2G1B*), x-prolyl aminopeptidase (*XPNPEP2*), Alpha-2-macroglobulin (*A2M*) and fatty acid binding protein 6 (*FABP6*) was repressed by 133.9, 70.6, 61.6, 58 and 45.8 folds, respectively. In addition, genes involved in steroid and lipid biosynthesis and metabolism, including hydroxysteroid (17-beta) dehydrogenase 7 (*HSD17B7*), hydroxy-delta-5-steroid dehydrogenase 3 beta- and steroid delta-isomerase 6 (*HSD3B6*), steroidogenic acute regulatory protein (*STAR*), 3-hydroxy-3-methylglutaryl-coenzyme A synthase 1 (*HMGCS1*), 3-hydroxy-3-methylglutaryl-coenzyme reductase (*HMGCR*) and cytochrome P450 (*CYP51, CYP11A1*, *CYP19A1*) were repressed in DHT-treated ovaries.

### Functional classification of the genes repressed in ovaries of DHT-treated rats

**Figure 3:**
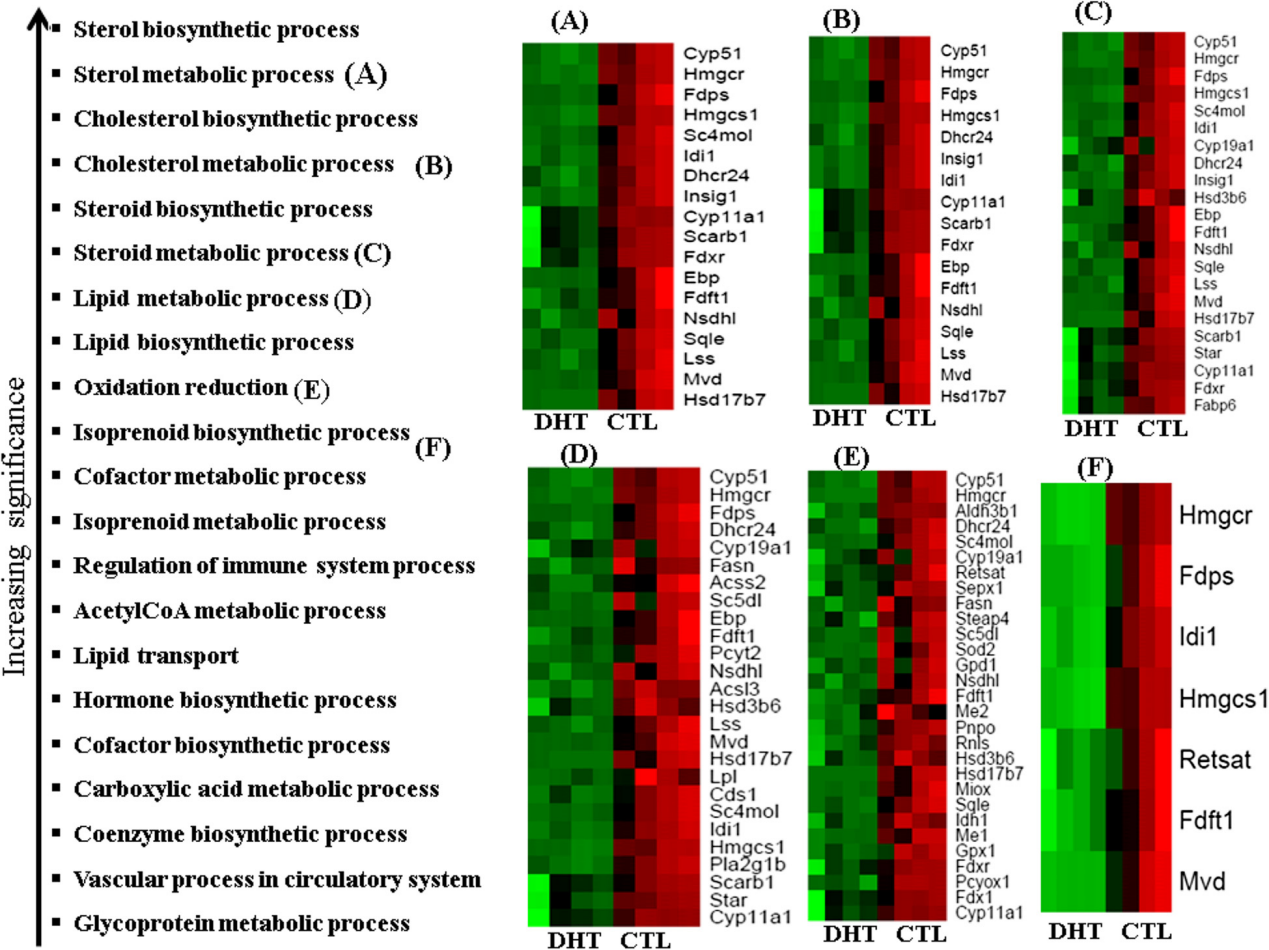
The top biological processes enriched by genes which were repressed in DHT-treated rat ovaries. The heatmaps on the right **(A, B, C, D, E, F)** describe clusters of genes involved in a particular function described by **A, B, C, D, E, F** on the left. The Heatmaps were generated using normalized log_2_ transformed values and the normalized log_2_ transformed expression values are described by pseudo color scale with red in CTL groups indicating the activated transcript level while the green color in DHT shows the repressed expression pattern of a specific gene. DHT represents biological replicates in DHT-treated ovaries while CTL represents biological replicates in rats which receive an empty silastic capsule.

**Figure 4:**
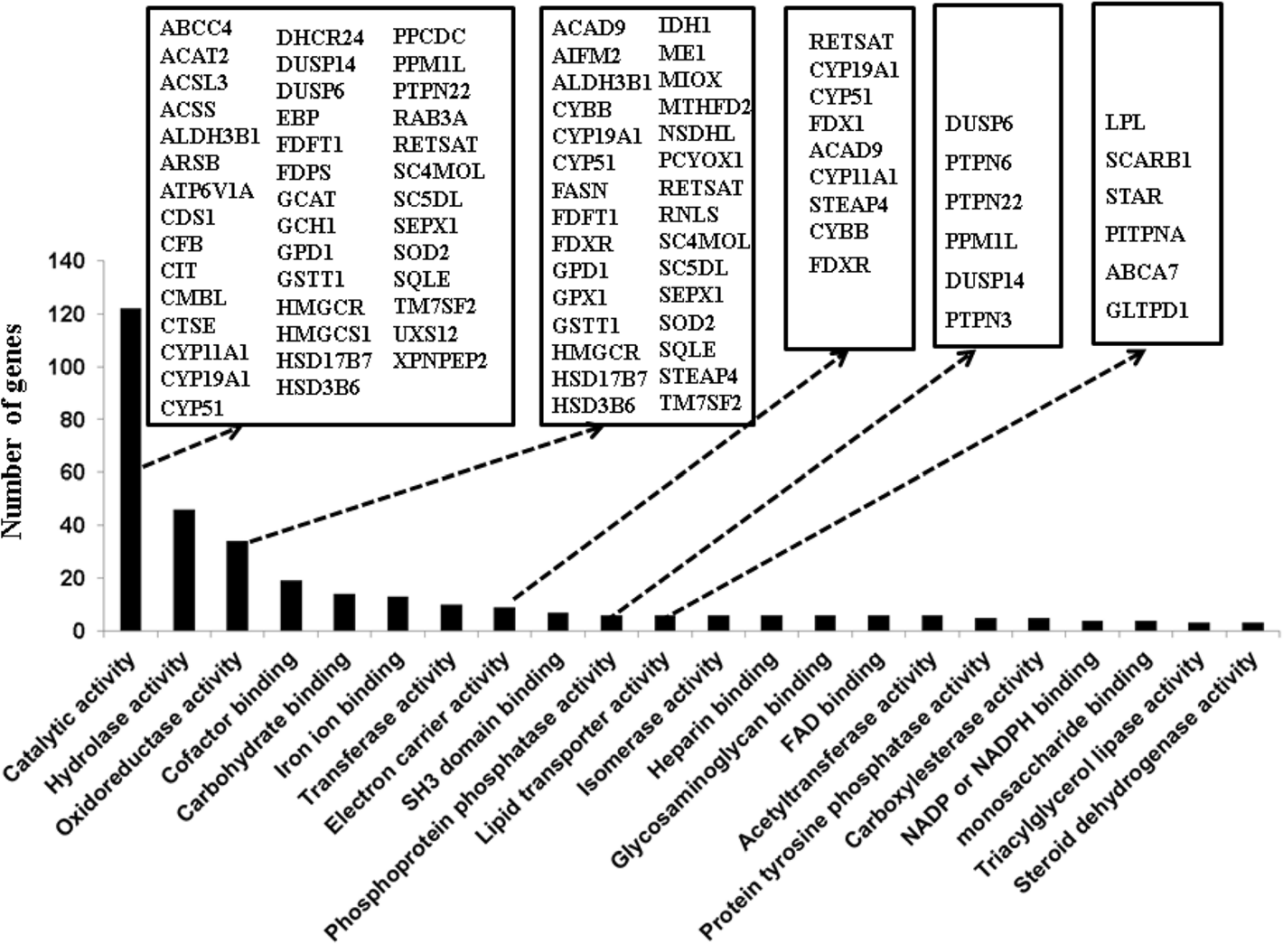
Molecular functions overrepresented by repressed genes in DHT-treated ovaries.

To understand the biological processes and molecular function over- or under-represented in ovaries associated with repressed genes in the DHT-treated rats, the functional annotation of the repressed genes were interrogated using the DAVID bioinfomatic tool and the result revealed a total 31 crucial biological processes to be repressed in DHT-treated ovaries (Additional file 1: Table S1). Most importantly, closely interlinked biosynthetic and metabolic functions, namely the biosynthesis and metabolism of sterols/steroids, cholesterol, isoprenoids and lipids, were the top significant biological processes enriched by repressed genes (Figure 3). In addition, the repressed genes were also found to be associated with oxidation/reduction, reactive oxygen species, metabolism and immune processes. Moreover, categorization of the repressed genes into their corresponding activities (molecular functions) revealed that 28% of them were known to be involved in catalytic activity, whereas 15% of the repressed genes are associated with binding of cofactors, coenzymes, irons, carbohydrates, SH3 domains, FADs, heparins, glycosaminoglycans, NADPs/NADPHs, cholesterols, glucoses, immunoglobulins and sterols (Figure 4, Additional file 2: Table S2). Moreover, 34 repressed genes, including *IDH1, ME1, NSDHL, FASN, GPD1, CYBB, FDXR, ALDH3B1* and *ACAD9,* are believed to regulate oxidoreductase activity (Figure 4, Additional file 2: Table S2).

### Gene transcripts activated in ovaries of DHT-treated rats

**Figure 5:**
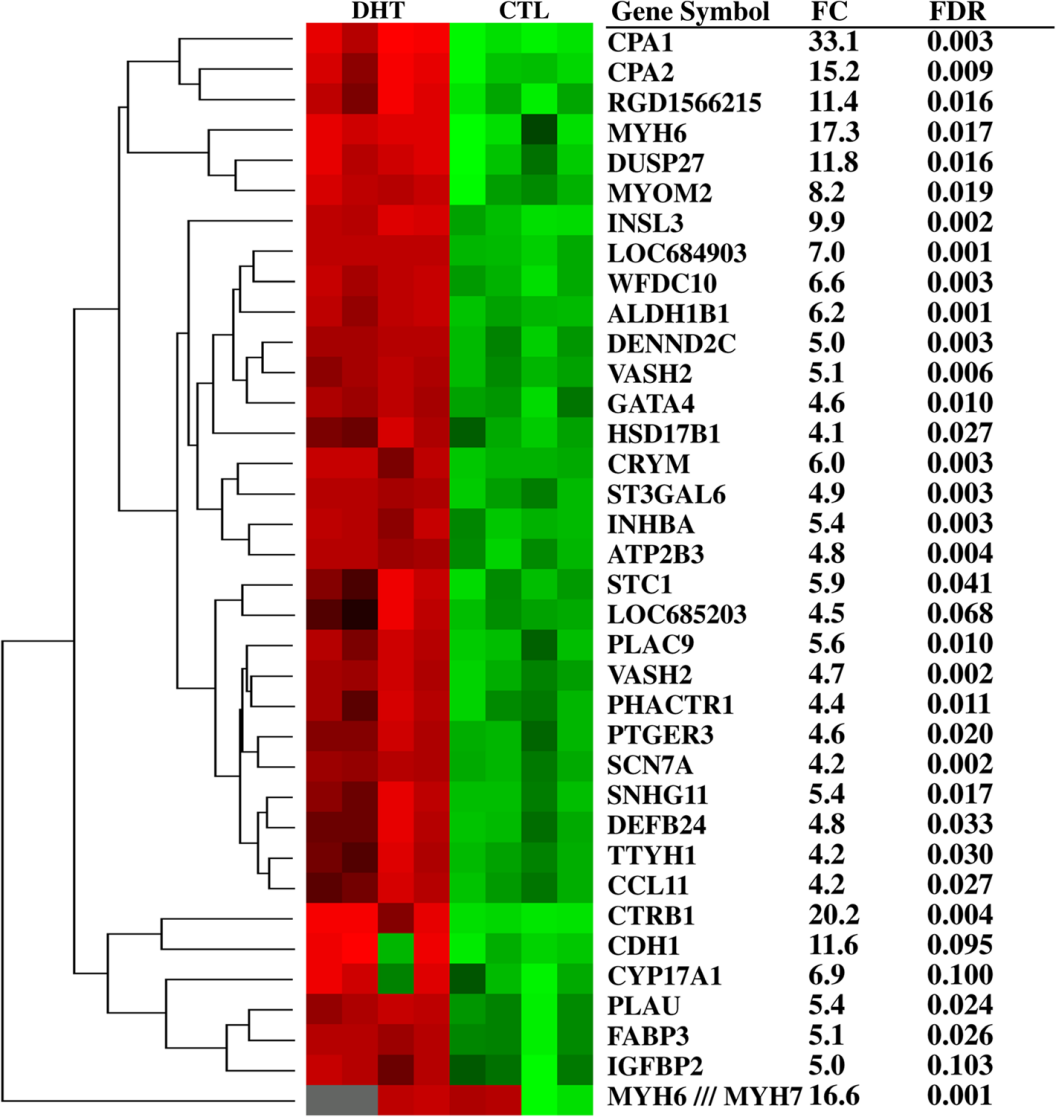
The hierarchical clustering and expression pattern of highly activated genes in DHT-treated ovaries relative to CTL group. DHT and CTL on the dendogram represent biological replicates in DHT-treated and CTL rat ovaries, respectively. The Heatmap was generated using normalized log_2_ transformed values and the normalized log_2_ transformed expression values are described by pseudo color scale with red in DHT-treated group indicating the activated transcript level while the green color in CTL group describes the repressed expression pattern of a specific gene.

To identify the transcripts activated in rat ovaries by DHT treatment, genes of which the expression was increased by ≥ 2-fold (p ≤ 0.01, FDR < 0.1) were investigated, using bioinformatic tools and literature mining. A total of 573 gene transcripts (57.1% of the total dysregulated genes) were activated in ovaries of DHT-treated rats. The list of top activated genes (with ≥ 4 fold changes) and their expression pattern within and between biological replicates is provided in Figure 5. The most activated genes (between 8- and 35-fold change) include carboxypeptidase A1 (*CPA1*), chymotrypsinogen B1 (*CTRB1*), myosin heavy chain (*MYH6*), carboxypeptidase A2 (*CPA2*), dual specificity phosphatase 27 (*DUSP27*) and cadherin 1 (*CDH1*). In addition, increased expression of genes involved in follicular growth and function was also evident in DHT-treated ovaries, including, anti-Mullerial hormone (*AMH)*, aldehyde dehydrogenase 1B1 (*ALDH1B1*), inhibins (*INHBA*, *INHBB*, *INHA*), *CYP17A1*, retinol binding protein (*RBP4*), growth arrest specific (*GAS6*, *GAS7*) and *GATA* binding protein 4 (*GATA4*).

### Molecular functions activated in ovaries of DHT-treated rats

**Figure 6:**
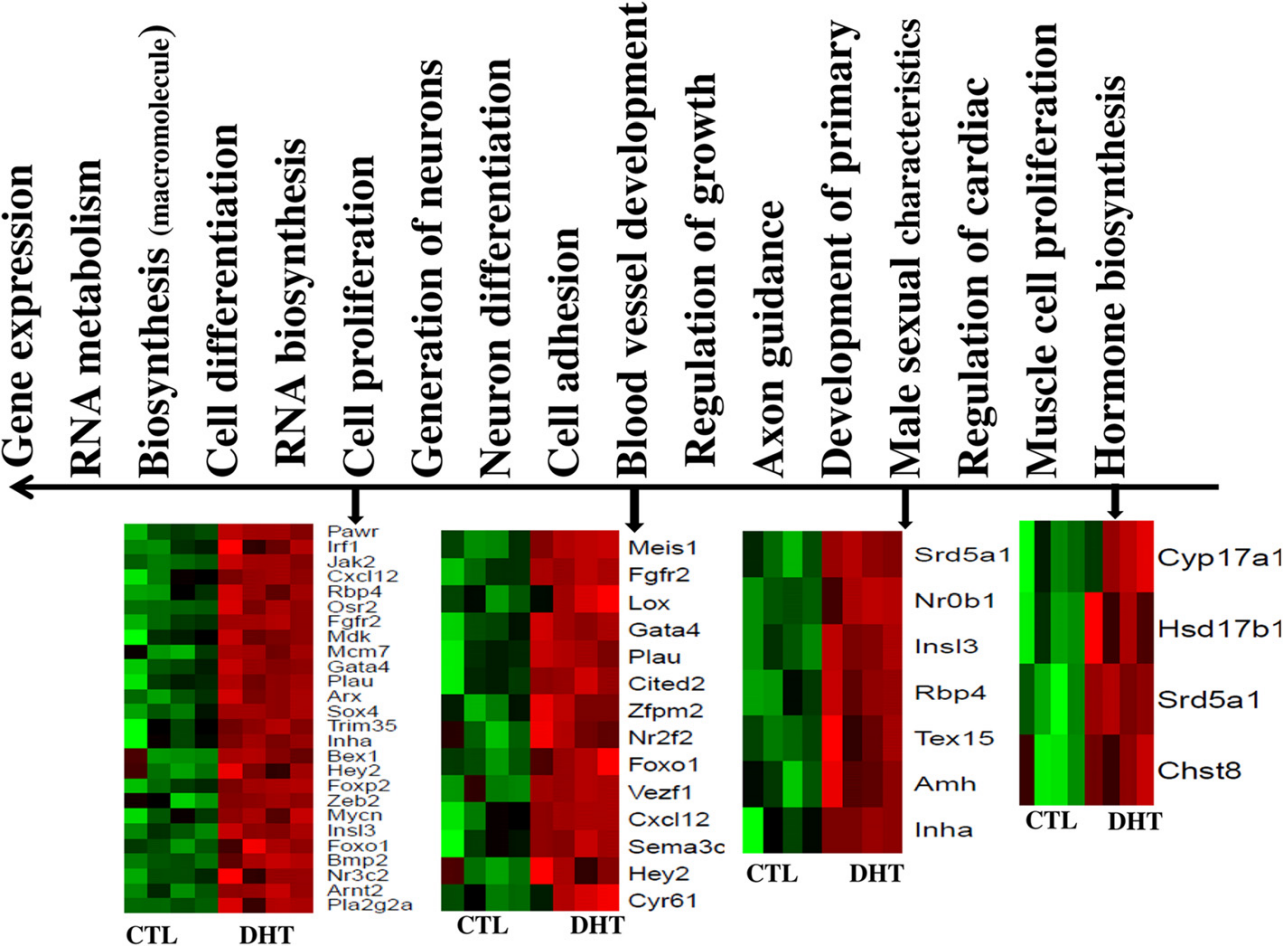
The most significantly molecular functions enriched by genes activated in DHT-treated ovaries. The direction of the arrow shows the significant level of the gene ontology terms. The heatmaps describe the expression pattern of clusters of genes involving in selected functions. Heatmaps were generated using normalized log2 transformed values and the normalized log2 transformed expression values are described by pseudo color scale with red in DHT group indicating the activated transcript level while the green color in CTL shows repressed expression pattern of a specific gene. DHT and CTL on the bottom of the heatmaps represent biological replicates in DHT-treated rat ovaries and rats which receive an empty silastic capsule, respectively

Gene set enrichment analysis showed 18 candidate biological processes, including cell differentiation/proliferation, transcriptional regulation/gene expression, neurogenesis, cell adhesion, RNA metabolism, macromolecule biosynthesis and blood vessel development processes to be affected in DHT-treated ovaries due to gene activation (Figure 6). Moreover, the activated genes known to be involved in selective and non-covalent binding of zinc ions, receptors, growth factors*,* protein phosphatases, DNA secondary structure and peptide antigens. In addition, some of the activated genes are known to be involved in growth factor activity, initiating for cell growth or proliferation and transcription corepressor activity (Table 2).

**Table 2:** **List of molecular functions containing genes with increased level of mRNA in DHT treated compared to CTL rat groups**

**Zinc ion binding**	***NR2F2,MAP3K1,PTGR1,CPA1,VEZF1,ACY3,SLC39A8,CPA2,RBM5,ZFP278,ZNF574, ZEB2, JARID1A, PHC1, ZCCHC11, KLF11, ZFPM2, OSR2, MSL2L1, TRIM37, PAN3, ZC3H11A, RGNEF, CRYZ, SIVA1, ZFP26, TRIM35, ZFP61, FOXP2, DTX3, ZNF292, GATA4, KLF15, RNF138, MMP23***
**Transcription regulator activity**	***NR2F2, CITED2, CRYM, ARNT2, NFIA, NR3C2, BMP2, SMARCD3, RPL7, MYCN, ZFP278, ZEB2, KLF11, ZFPM2, ARX, TLE1, FOXP2, ZNF292, GATA4, NR0B1,RBPJ, MEIS1, HOXD9, CDH1, FOXO1,TWIST, KLF15***
**Receptor binding**	***INSL3, INHA, JAK2, AKAP9, CXCL12, INHBB, ARNT2, INHBA, PENK, BMP2,SMARCD3, SEMA3C, EPHA4,SEMA6A, SIVA1, NR0B1, GAS6, ANGPTL1, MDK,STC1, IRS3***
**Protein dimerization activity**	***INHA, INHBB, GHR, ARNT2, NR3C2, SHMT1, INHBA, BMP2, MYH6, MYH7, RPL7, PON3, GUCY1A3, FOXP2, NR0B1, MEIS1, ROBO2***
**Growth factor activity**	***INHA, JAK2, INHBB, INHBA, BMP2, GAS6, MDK***
**Carboxylic acid transmembrane transporter activity**	***SLCO1A4, SLC13A5, SLC1A3, SLC7A5, SLC7A8***
**Growth factor binding**	***FGFR2, IGFBP6, CRIM1,HTRA3, CYR61***
**Insulin-like growth factor binding**	***IGFBP6, CRIM1, HTRA3, CYR61***
**Protein phosphatase binding**	***GHR, PHACTR1, JUP, CDH1***
**Extracellular matrix binding**	***VTN, RPSA, CYR61***
**L-amino acid transmembrane transporter activity**	***SLC1A3,SLC7A5, SLC7A8***
**Semaphorin receptor binding**	***SEMA3C, SEMA6A***
**Protein tyrosine phosphatase-like protein binding**	***JUP, CDH1***
**Actin-dependent atpase activity**	***MYH6, MYH7***
**Glycine hydroxyl methyltransferase activity**	***SHMT1, GART***
**Peptide antigen binding**	***SLC7A5, SLC7A8***
**Metallocarboxypeptidase activity**	***CPA1, CPA2***

### Molecular pathways activated or repressed in DHT-treated rats

In addition to their biological or molecular functions, an important and significantly dysregulated molecular interactions and relations associated with the activated or repressed genes were identified using KEGG and Panther gene enrichment analysis. The studies suggest 19 molecular pathways being affected in DHT-treated group (Table 3). Among these, cholesterol and trepenoid biosynthesis pathways, citrate cycle, androgen and estrogen metabolism were enriched by repressed genes while several metabolic pathways including glycolysis/gluconeogenesis, fatty acid metabolism, pyruvate metabolism, butanoate metabolism, lysine, leucine, valine and isoleucine degradation and glutathione metabolic pathways were enriched by both activated and repressed genes (Table 3).

**Table 3:** **List of molecular pathways containing genes with activated (↑) and repressed (↓) level of mRNA in DHT treated compared to CTL rat ovary groups**

**Biosynthesis of steroids**	**↓** ***CYP51***, ↓***HMGCR***, ↓***SQLE***, ↓***HSD17B7***, ↓***FDFT1***, ↓**LSS**, ***MVD***, ↓***FDPS***, ↓***IDI1***, ↓***SC5DL***, ↓***EBP***, ↓***SC4MOL***, ↓***TM7SF2***, ↓***DHCR24***, ↓***NSDHL***
**TGF-beta signaling pathway**	***↑INHBB***, ***↑INHBA***, ***↑AMH***, ***↑BMP2***, ***↑FOXO1***, ***↑FOXP2***, ↓***FKBP1A***,***↑ INHA***, ***↑*** **BAMBI**, ***↑CITED1***, ↓***CITED2***
**Leukocyte transendothelial migration**	***↑CXCL12***, ↓***THY1***, ↓***BCAR1***, ↓***PECAM1***,↓ ***CXCR4***, ***↑CLDN11***, ***↑MYL9***, ↓***SIPA1***
**Cholesterol biosynthesis**	↓***MVD***, ↓***HMGCR***, ↓***HMGCS1***, ↓***FDPS***, ↓***IDI1***, ↓***FDFT1***
**Pyruvate metabolism**	↓***ME1***, ↓***DLAT***, ***↑ALDH1B1***, ↓***ME2***, ↓***ACAT2***, ↓***ACSS2***, ***↑ME3***
**Glycerophospholipid metabolism**	↓***PLA2G1B***, ***↑PLA2G2A***, ↓***GPD1***, ↓***CDS1***,↓ ***PCYT2***, ***↑ETNK2***, ***↑CRLS1***
**Complement and coagulation cascades**	↓***A2M***, ↓***C2***, ↓***PLAU***, ***↑TFPI***, ***↑MASP1***,↓ ***CFB***, ↓***C1QA***
**Glycolysis/Gluconeogenesis**	↓***HK1***, ↓***HK2***, ↓***DLAT***, ***↑FBP2***, ***↑ALDH1B1***,↓***ACSS2***
**Adipocytokine signaling pathway**	***↑JAK2***, ↓***NFKBIB***, ***↑IRS3***, ↓***ACSL3***, ↓***ADIPOR***, ***↑STK11***
**Butanoate metabolism**	↓***HMGCS1***, ↓***AACS***, ***↑ALDH1B1***, ↓***ACAT2***, ***↑ACSM5***
**Terpenoid biosynthesis**	↓***SQLE***, ↓***FDFT1***, ↓***FDPS***, ↓**IDI1**
**Ether lipid metabolism**	↓***PLA2G1B***, ***↑PLA2G2A***, ***↑PAFAH1B3***, ↑***ENPP6***
**Citrate cycle (TCA cycle)**	↓***ACLY***, ↓***IDH1***, ↓***DLAT***, ↓***DLST***
**Androgen and estrogen metabolism**	***↑SRD5A1***, ↓ ***HSD17B1***, ↓***HSD17B7***, ↓***HSD3B6***
**Androgen/estrogene/progesterone biosynthesis**	↓***HSD3B6***, ↓***CYP11A1***, ↓***NSDHL***, ↓***CYP19A1***
**Glutathione metabolism**	↓***GPX1***, ↓***IDH1***, ↓***GSTT1***, ***↑GPX7***
**Lysine degradation**	***↑ALDH1B1***, ↓***DLST***, ↓***ACAT2***, ***↑MGC109340***
**Fatty acid metabolism**	***↑ACSL3***, ↓***ACAA2***, ***↑ALDH1B1***, ↓***ACAT2***
**Valine, leucine and isoleucine degradation**	↓***HMGCS1***, ↓***ACAA2***, ***↑ALDH1B1***, ↓***ACAT2***

### Validation of microarray data using real time quantitative PCR (qPCR)

To validate the expression data generated by microarray analysis, the expression level of 5 activated (*CTRB1*, *MYH6*, *PLAU*, *ST3GAL6* and *INHBA*) and 4 repressed (*STAR*, *HSD17B7*, *OSGIN1* and *RLN1*) genes were assessed by qPCR. The validation result shows that all the randomly selected genes displayed a similar trend to the microarray data, thus confirming the validity of the microarray results (Table 4).

**Table 4:** **The array and qPCR results for selected differentially expressed genes**

**Gene name**	**Gene symbol**	**Acess. No**	**Array result**		**qPCR result**	
			**FC**	**P value**	**FC**	**P value**
**Chymotrypsinogen b1**	**CTRB1**	**NM_012536**	**20 (↑)**	**0.00003**	**27.3 (↑)**	**0.0023**
**Myosin, heavy chain 6, cardiac muscle, alpha**	**MYH6**	**NM_017239**	**17 (↑)**	**0.0004**	**14 0 (↑)**	**0.06**
**Plasminogen activator, urokinase**	**PLAU**	**NM_013085**	**5.37 (↑)**	**0.0008**	**5.56 (↑)**	**0.13**
**St3 beta-galactoside alpha-2,3-sialyltransferase 6**	**ST3GAL6**	**NM_207602**	**4.9 (↑)**	**0.0126**	**5.28 (↑)**	**0.05**
**Inhibin beta-a**	**INHBA**	**NM_017128**	**5.43(↑)**	**0.002**	**28.9 (↑)**	**0.04**
**Steroidogenic acute regulatory protein**	**STAR**	**NM_031558**	**14.57 (↓)**	**0.001**	**25.5 (↓)**	**0.0018**
**Hydroxysteroid (17-beta) dehydrogenase 7**	**HSD17B7**	**NM_017235**	**30.52 (↓)**	**0.0003**	**47.67 (↓)**	**0.006**
**Oxidative stress induced growth inhibitor 1**	**OSGIN1**	**NM_138504**	**26.98 (↓)**	**0.000022**	**21.57 (↓)**	**0.0047**
**Relaxin 1**	**RLN1**	**NM_013413**	**133.9 (↓)**	**0.0014**	**255.8 (↓)**	**0.019**

FC = Fold change, ↑ and ↓ indicate the activated and repressed genes in DHT treated compared to CTL rat groups, p ≤ 0.05 considered as significant.

## Discussion

Using a rat PCOS model, we have previously reported altered ovarian expression pattern of 83 miRNAs following DHT treatment [23]. In this study, we investigated the gene expression profile of the same ovarian samples and identified 573 activated and 430 repressed gene transcripts in DHT-treated rats, suggesting the presence of transcriptome profile dysregulation due to hyperandrogenism. In addition, the cellular localization of the products of the activated or repressed gene showed 180 of the dysregulated ones were present in the nucleus while the majority were localized in the cytoplasm (Additional file 3: Figure S1), suggesting a possible dysregulation of genes function associated with specific ovarian subcellular localization.

The number of dysregulated ovarian genes in the DHT-treated rats appeared consistent with an earlier report by human ovarian cDNA microarray [30] in which the number of up-regulated genes (n = 88) was relatively higher than the down-regulated ones (n = 31) in ovaries of PCOS women compared to the non-PCOS subjects. However in another study where the GeneChips HG_U133A and HG_U133B arrays from Affymetrix used, majority of the dysregulated genes in PCOS were found to be down-regulated [31]. Although the reasons for these apparent discrepancies are not clear, the possibility that this could be due to the differences in tissue sampling, the microarray platform used and the statistical analysis cannot be excluded. To evaluate whether altered ovarian genes in DHT-treated rat resemble those of PCOS women, we merged our data with the supplemental data previously reported [31] (http://press.endocrine.org/doi/suppl/10.1210/me.2004-0074/suppl_file/suppltable1part1me_04_0074.xls) and noted that 160 dysregulated genes were observed in ovaries of both DHT-rats and PCOS women (Additional file 4: Table S3).

In the current study, we have described the key biological processes, molecular functions and pathways affected by dysregulated genes in DHT-treated rats. While the text mining approach would shed light on the relevance of the dysregulated genes with respect to the PCOS phenotype, the present global approach involving pathway analysis and molecular functions provided a more comprehensive understanding of the potential genetic mechanism underling PCOS phenotypes. Our findings also demonstrated ovarian cell type-specific changes in expression of genes involved in granulosa cell proliferation and progesterone biosynthesis [*AMH*, *RBP4* and cytochrome P450s (*CYP51, CYP19A1, CYP11A1* and *CYP17A1*) and dysregulation of the genes associated with cholesterol biosynthesis and metabolism (acetyl-CoA acetyltransferases enzymes, *ACTAs* and *HMGCR*) in DHT-treated ovaries. These findings are consistent with the dependence of granulosa cell progesterone biosynthesis on the de novo cholesterol synthesis [32]. More importantly, the action of *HMGCR* is believed to be the rate limiting step in cholesterol biosynthesis [33,34].

**Figure 7:**
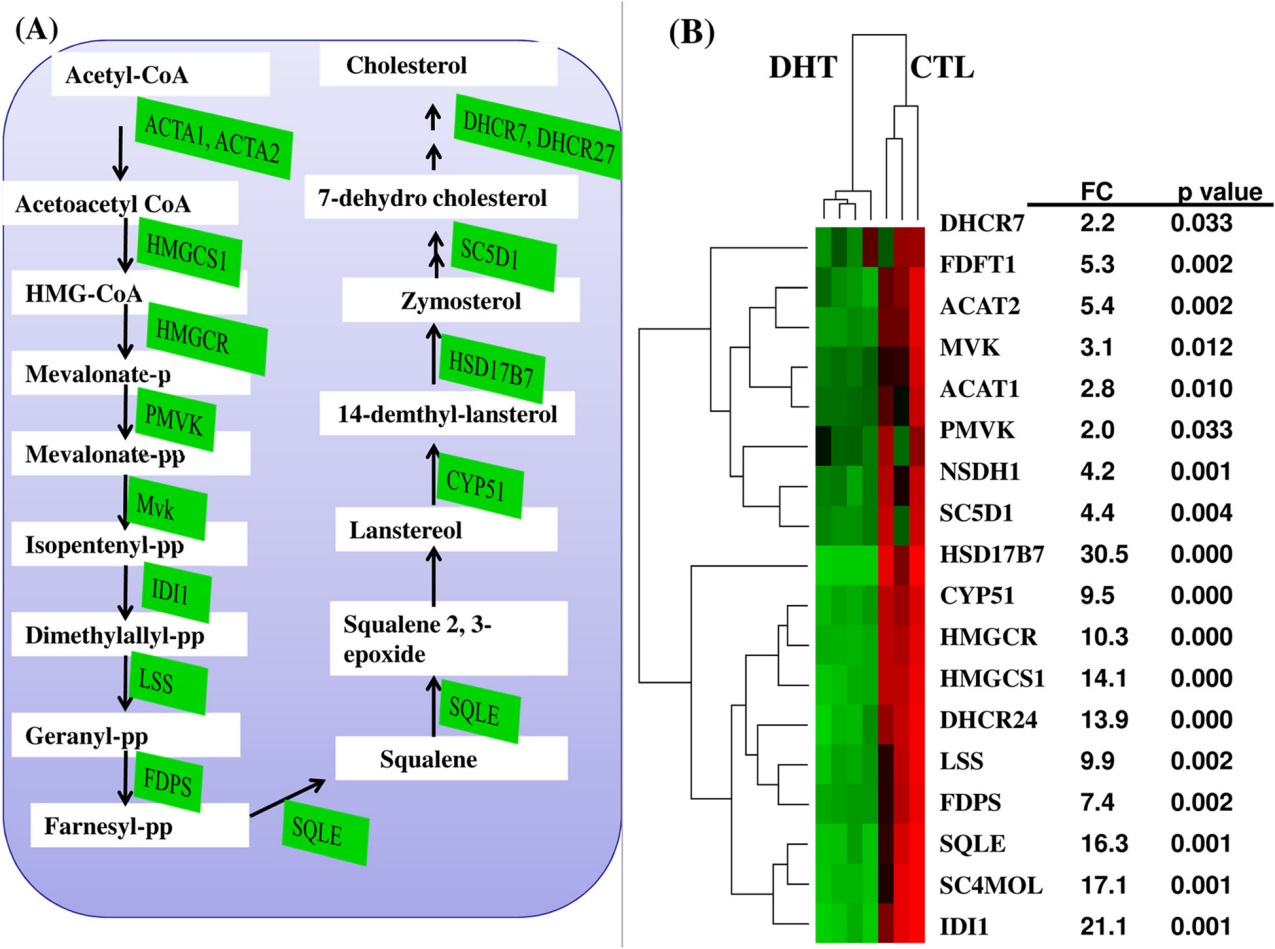
The expression pattern of genes involving in cholesterol biosynthesis pathway in DHT-treated rat ovaries. **(A)** A modified cholesterol biosynthesis pathway outlined by [51,52] and the intermediate and final products performed by repressed genes in DHT-treated ovaries. The genes are indicated in green box while the intermediate products are described in white box. **(B)** The expression pattern of genes involving in cholesterol biosynthesis in DHT-treated ovaries compared to CTL. FC: The average fold change reduction in DHT compared to CTL. P value: the significant level. The red color indicates the activated transcript in CTL level while the green color shows the repressed expression pattern of a specific gene in DHT rat groups.

Our present findings suggest that the dysregulation of the isoprene pathway in cholesterol biosynthesis, leading to abnormal accumulation of cholesterol precursors in hyperandrogenic state. Moreover, down-regulation of cholesterol biosynthesis may limit the amount of cholesterol influx and in turn affect ovarian cellular functions including fertility in DHT-treated rats. Here, a series of the enzymes involving in the conversion of mevalonate to arnesyl-diphosphate, isoprepene to saquale, and squalene in cholesterol were also repressed in ovaries of DHT-treated rats (Figure 7).

**Figure 8:**
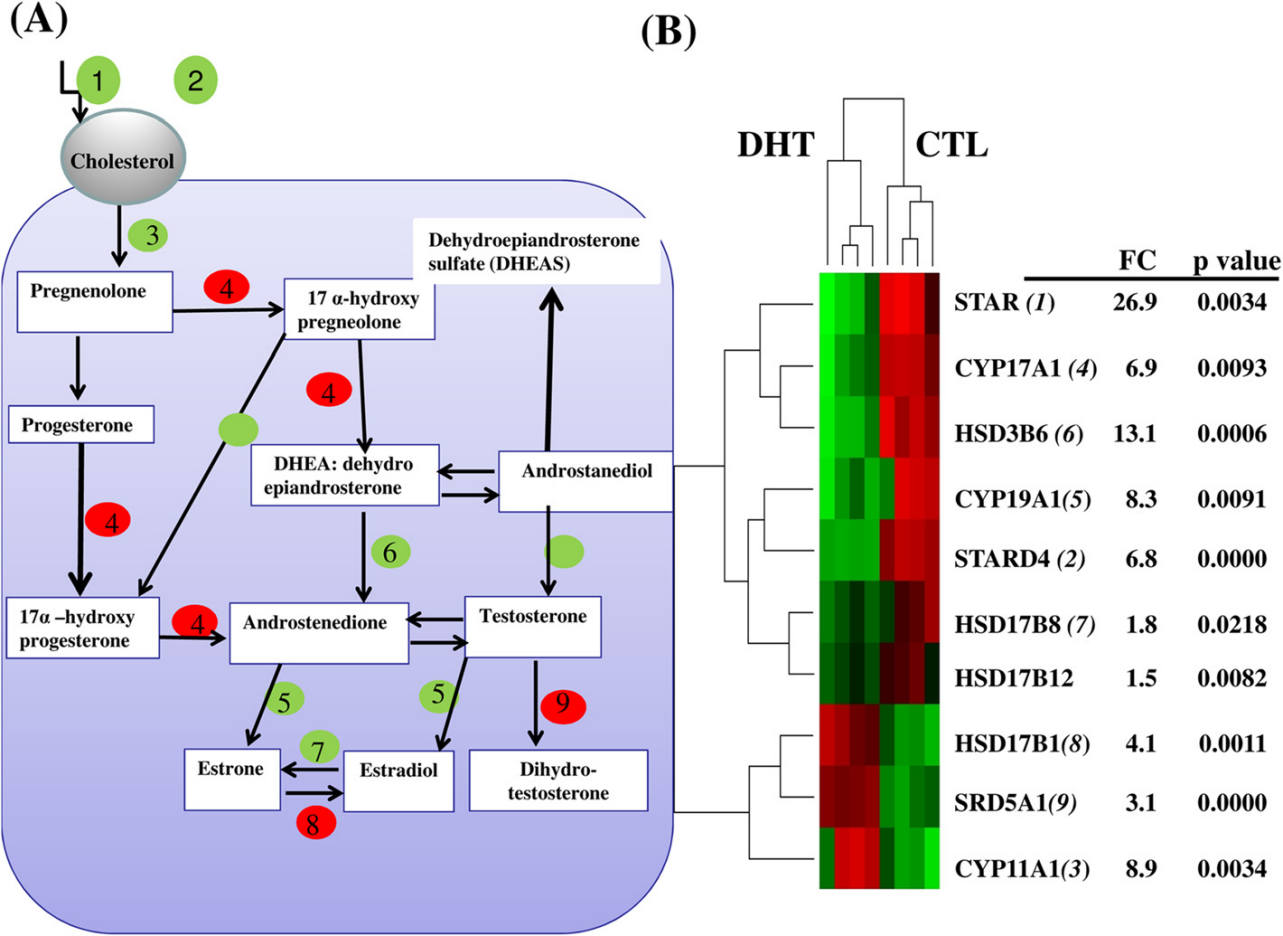
The expression pattern of genes involving in sex steroid biosynthesis pathway in DHT-treated rat ovaries. **(A)** A modified steroid biosynthesis pathway from [53]. The intermediate and final products performed by dysregulated genes in DHT-treated ovaries are indicated in white box while the number in red or green circle corresponds the genes (enzymes) indicated in Figure B. **(B)** The heatmap displaying the expression pattern of repressed and activated genes involving in steroid biosynthesis in DHT compared to CTL. FC indicates the average fold change gene expression reduction or activation in DHT compared to CTL. P value: the significant levels.

One of the major functions of the ovary is the production of steroid hormone [35]. Here, we identified dysregulation of several genes associated with steroidogenesis in ovaries of DHT-treated rats, including repression of cytochrome P450s*,* STAR and 3β-hydroxysteroid dehydrogenases (Figure 8). The repression of STAR in DHT-treated group suggests a reduced level of cholesterol influx for downstream steroidogenesis. In addition, the down-regulation of *CYP11A1* and CYP19A1 in DHT-treated ovaries was consistent with our previous publication showing decreased aromatase expression and estradiol secretion in granulosa cells from DHT-treated rats [36]. Therefore, the dysregulation of genes associated with steroidogenesis in DHT-treated rats could result in abnormal sex hormone levels and ultimately PCOS phenotypes, including cycle irregularity, abnormal folliculogenesis and anovulation.

Women with PCOS may have higher abdominal body fat distribution, due to hyperandrogenism and insulin resistance [37]. This phenomenon may be attributed by increased level of lactate, long-chain fatty acids and triglyceride [38-40]. We have previously demonstrated that the rats treated with DHT exhibited higher body weight compared to control [23]. Although data regarding the total fat content of the ovary is lacking, examination into the gene set enrichment analysis revealed 42 genes associated with lipid metabolism and biosynthesis were repressed in DHT-treated rats. Among those, 26 genes including acyl-CoA synthetase and fatty acid synthase are known to be involved in dual roles of lipid synthesis and metabolism, while 16 other genes were related only to lipid metabolism. The dysregulation of genes involving *de novo* lipid synthesis and metabolism in the DHT group may result in the accumulation of lipid precursors or lack of essential fatty acids which are required for normal ovarian physiology.

One of the characteristics of PCOS is the presence of atretic follicles or premature growth arrest without atresia [41,42]. These phenotypic manifestations could be due to defects in steroid biosynthesis and energy metabolism. In line with this notion, excess androgen, luteinizing hormone and insulin are associated with the recruitment of several but small preovulatory follicles [5]. Indeed, cell cycle progression and proliferation is thought to be controlled by several regulators [43]. In the current study, a total of 45 genes that associated with cell proliferation and differentiation, including *AMH* and *BMP2*, were activated in ovaries of DHT-treated rats (Figure 6). *AMH* inhibits primordial follicle recruitment and decreases the sensitivity of growing follicles to FSH [44]. *AMH* nulls and heterozygous mice exhibited early depletion of primordial follicles [45]. Similarly, it is possible that *BMP2* gene activated in DHT-treated ovaries could participate in the regulation of folliculogenesis and luteinization by modulating gonadotropin receptor expression [46]. Activation of these genes may induce small follicle growth but dominant follicle growth arrest in DHT-treated rats, although this possibility needs further investigation.

In addition, genes involved in glycolysis/gluconeogenesis are also dysregulated by DHT treatment. It is known that hexokinases (HK1/2) convert glucose to glucose 6-phosphate [47] while pyruvate dehydrogenase complex component x (*PDHX)* catalyzes the conversion of pyruvate to acetyl coenzyme A [48,49]. In our study, ovarian *HK1*, *HK2*, *PDHX* and *ACSS2* were repressed but *FBP2* and *ALDH1B1* were activated in DHT-treated rats (Table 3), further complicating the metabolic disorders in those groups. This finding is consistent with earlier report indicating the down-regulation of several genes regulating glucose synthesis and consumption in PCOS patients [50]. In addition, down-regulation of the oxidative reductase gene (Figure 3E) and those of citrate acid cycle pathway (Table 3) adds further evidence for dysregulated energy metabolism in DHT-treated ovaries.

In conclusion, we have provided detailed evidence for transcriptome profile changes in a chronically androgenized PCOS rat model. Our data suggest biosynthesis and metabolism of cholesterol, sterols/steroids, lipids and oxidation/reduction are key molecular functions associated with repressed gene expression in DHT-treated rats. On the other hand, cell differentiation/proliferation, transcriptional regulation, neurogenesis, cell adhesion and blood vessel development were enriched by activated genes in this animal model. It is therefore conceivable that these molecular functional alterations could be a molecular clue for abnormal steroidogenesis, estrous cycle irregularity, abnormal folliculogenesis, anovulation, and disorders in carbohydrate regulation and lipid metabolism occurring in PCOS patients. This study contributes significantly to our understanding of the ovarian transcriptome profile and associated molecular functional alterations in DHT-treated rats, and provides the basis for future in-depth functional and mechanistic studies that to shed light on the pathophysiologic significance of the current findings in PCOS.

## Additional files

Additional file 1: Table S1. Significant biological processes enriched by genes repressed in DHT-treated ovaries. Additional file 2: Table S2. Significant molecular functions enriched by genes repressed in DHT-treated ovaries. Additional file 3: Figure S1. Cellular localization (cellular components) of dysregulated genes in DHT- treated ovaries. Additional file 4: Table S3. The expression pattern of some dysregulated genes in DHT rat ovaries in the current data set and their corresponding expression level in PCOS human ovary and long-term androgen-treated female-to-male transsexuals (TSX) compared to healthy ovaries [31].
