# Supplementary material for: Polycystic ovarian syndrome is accompanied by repression of gene signatures associated with biosynthesis and metabolism of steroids, cholesterol and lipids
Source: J Ovarian Res. 2015 Apr 13;8:24. doi: 10.1186/s13048-015-0151-5 (PMC4414284; doi:10.1186/s13048-015-0151-5)
Supplement: Additional file 1: Table S1. — Significant biological processes enriched by genes repressed in DHT-treated ovaries. [file 13048_2015_151_MOESM1_ESM.docx]

Table S1. Ssignificant biological processes enriched by genes repressed in

DHT treated ovaries.

| GOBPID | Pvalue | Count | Size | Term |
| --- | --- | --- | --- | --- |
| GO:0016126 | 3.08E-14 | 13 | 28 | Sterol biosynthetic process |
| GO:0016125 | 6.01E-14 | 18 | 70 | Sterol metabolic process |
| GO:0006695 | 1.88E-13 | 12 | 25 | Cholesterol biosynthetic process |
| GO:0008203 | 2.99E-13 | 17 | 66 | Cholesterol metabolic process |
| GO:0006694 | 3.63E-13 | 18 | 77 | Steroid biosynthetic process |
| GO:0008202 | 5.16E-11 | 22 | 158 | Steroid metabolic process |
| GO:0006629 | 1.73E-10 | 42 | 570 | Lipid metabolic process |
| GO:0008610 | 3.41E-10 | 26 | 243 | Lipid biosynthetic process |
| GO:0006066 | 1.46E-09 | 27 | 279 | Cellular alcohol metabolic process |
| GO:0055114 | 1.03E-07 | 29 | 385 | Oxidation reduction |
| GO:0008299 | 6.04E-07 | 6 | 14 | Isoprenoid biosynthetic process |
| GO:0051186 | 8.50E-07 | 17 | 166 | Cofactor metabolic process |
| GO:0006720 | 1.15E-05 | 7 | 32 | Isoprenoid metabolic process |
| GO:0032787 | 0.0003211 | 17 | 262 | Monocarboxylic acid metabolic process |
| GO:0002682 | 0.00114071 | 17 | 293 | Regulation of immune system process |
| GO:0006084 | 0.00129053 | 5 | 33 | Acetyl-coa metabolic process |
| GO:0006869 | 0.0012938 | 8 | 85 | Lipid transport |
| GO:0042446 | 0.00218454 | 5 | 37 | Hormone biosynthetic process |
| GO:0051188 | 0.00250842 | 7 | 74 | Cofactor biosynthetic process |
| GO:0010035 | 0.00740892 | 10 | 161 | Response to inorganic substance |
| GO:0019752 | 0.01016314 | 20 | 453 | Carboxylic acid metabolic process |
| GO:0006082 | 0.01063569 | 20 | 455 | Organic acid metabolic process |
| GO:0009108 | 0.01210771 | 5 | 55 | Coenzyme biosynthetic process |
| GO:0003018 | 0.01355921 | 6 | 78 | Vascular process in circulatory system |
| GO:0042221 | 0.02487924 | 37 | 1064 | Response to chemical stimulus |
| GO:0009100 | 0.02695364 | 6 | 91 | Glycoprotein metabolic process |
| GO:0003006 | 0.03162836 | 7 | 120 | Reproductive developmental process |
| GO:0048666 | 0.03272748 | 14 | 322 | Neuron development |
| GO:0042110 | 0.04601073 | 8 | 158 | T cell activation |
| GO:0007409 | 0.04656061 | 9 | 187 | Axonogenesis |
| GO:0000302 | 0.04857745 | 5 | 79 | Response to reactive oxygen species |
