# Supplementary material for: Polycystic ovarian syndrome is accompanied by repression of gene signatures associated with biosynthesis and metabolism of steroids, cholesterol and lipids
Source: J Ovarian Res. 2015 Apr 13;8:24. doi: 10.1186/s13048-015-0151-5 (PMC4414284; doi:10.1186/s13048-015-0151-5)
Supplement: Additional file 2: Table S2. — Significant molecular functions enriched by genes repressed in DHT-treated ovaries. [file 13048_2015_151_MOESM2_ESM.docx]

Table S2. Significant molecular functions enriched by genes repressed in

DHT treated ovaries.

| GOMFID | Pvalue | Count | Size | Term |
| --- | --- | --- | --- | --- |
| GO:0003824 | 2.88E-06 | 122 | 3443 | catalytic activity |
| GO:0016787 | 0.03901096 | 46 | 1412 | hydrolase activity |
| GO:0016491 | 4.27E-07 | 34 | 529 | oxidoreductase activity |
| GO:0048037 | 1.32E-06 | 19 | 209 | cofactor binding |
| GO:0050662 | 2.63E-07 | 17 | 152 | coenzyme binding |
| GO:0030246 | 0.00207664 | 14 | 230 | carbohydrate binding |
| GO:0005506 | 0.004292 | 13 | 223 | iron ion binding |
| GO:0016746 | 0.00095439 | 10 | 121 | transferase activity, transferring acyl groups |
| GO:0009055 | 0.01427195 | 9 | 151 | electron carrier activity |
| GO:0005529 | 0.02180615 | 8 | 136 | sugar binding |
| GO:0008415 | 0.00697118 | 8 | 111 | acyltransferase activity |
| GO:0017124 | 0.00048543 | 7 | 56 | SH3 domain binding |
| GO:0030247 | 0.0075857 | 7 | 90 | polysaccharide binding |
| GO:0001871 | 0.01186436 | 7 | 98 | pattern binding |
| GO:0004721 | 0.04592353 | 6 | 103 | phosphoprotein phosphatase activity |
| GO:0005319 | 0.00137372 | 6 | 49 | lipid transporter activity |
| GO:0016853 | 0.02485904 | 6 | 89 | isomerase activity |
| GO:0008201 | 0.00390633 | 6 | 60 | heparin binding |
| GO:0005539 | 0.01927422 | 6 | 84 | glycosaminoglycan binding |
| GO:0050660 | 0.00358991 | 6 | 59 | FAD binding |
| GO:0016407 | 0.00152862 | 6 | 50 | acetyltransferase activity |
| GO:0004725 | 0.02832029 | 5 | 68 | protein tyrosine phosphatase activity |
| GO:0004091 | 0.01522338 | 5 | 58 | carboxylesterase activity |
| GO:0016790 | 0.04430757 | 4 | 53 | thiolester hydrolase activity |
| GO:0050661 | 0.00382897 | 4 | 26 | NADP or NADPH binding |
| GO:0048029 | 0.02095232 | 4 | 42 | monosaccharide binding |
| GO:0004806 | 0.00295478 | 3 | 12 | triacylglycerol lipase activity |
| GO:0016229 | 0.02694164 | 3 | 26 | steroid dehydrogenase activity |
| GO:0016417 | 0.00015318 | 3 | 5 | S-acyltransferase activity |
| GO:0016877 | 0.00698061 | 3 | 16 | ligase activity, forming carbon-sulfur bonds |
| GO:0016863 | 0.00051629 | 3 | 7 | intramolecular oxidoreductase activity, transposing C=C bonds |
| GO:0016860 | 0.03272453 | 3 | 28 | intramolecular oxidoreductase activity |
| GO:0016830 | 0.04605763 | 3 | 32 | carbon-carbon lyase activity |
| GO:0016408 | 0.00051629 | 3 | 7 | C-acyltransferase activity |
| GO:0016878 | 0.00225793 | 3 | 11 | acid-thiol ligase activity |
