# Supplementary figures and images for: Polycystic ovarian syndrome is accompanied by repression of gene signatures associated with biosynthesis and metabolism of steroids, cholesterol and lipids
Source: J Ovarian Res. 2015 Apr 13;8:24. doi: 10.1186/s13048-015-0151-5 (PMC4414284; doi:10.1186/s13048-015-0151-5)

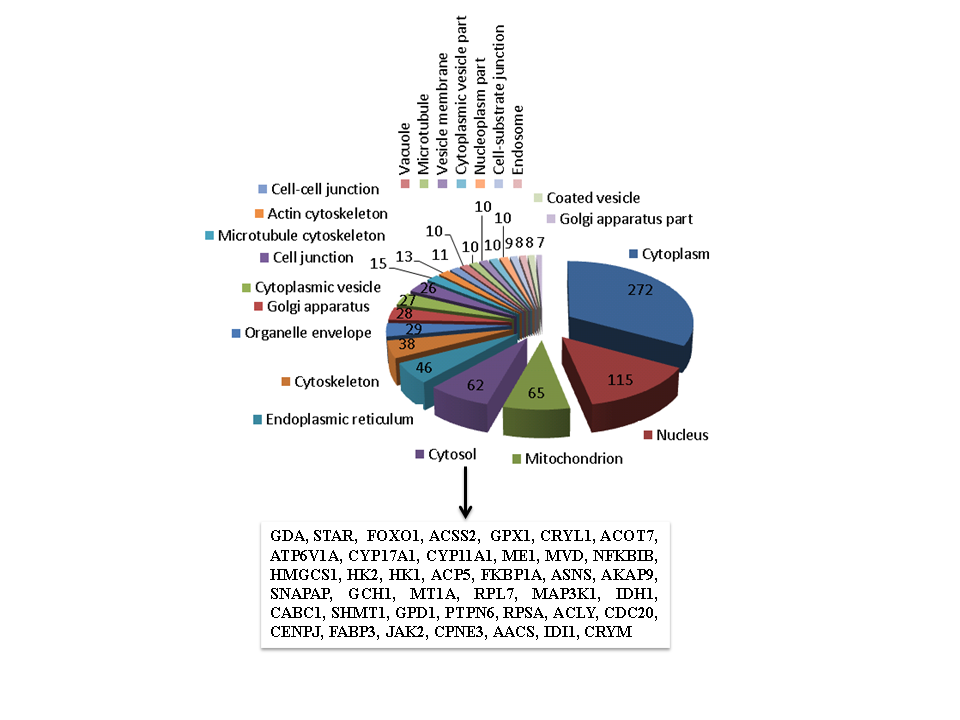

Supplement: Additional file 3: Figure S1. — Cellular localization (cellular components) of dysregulated genes in DHT- treated ovaries. [file 13048_2015_151_MOESM3_ESM.tiff]
